# Richard Turkington and Christopher Watson (eds.): Renewing Europe’s housing: Policy Press, UK, 2015, 328 pp, £70.00 (hardback), ISBN: 978-1-44731-012-9.

**DOI:** 10.1007/s10901-016-9520-7

**Published:** 2016-07-26

**Authors:** André Thomsen

**Affiliations:** Delft University of Technology, Delft, The Netherlands

*Renewing Europe’s Housing* provides an extensive overview of contemporary housing renewal policies and practices in Europe during the last four decades.

The subject is of high and still increasing importance. During the time covered by the book, housing and urban policies in most European countries shifted thoroughly. After half a century of unsurpassed mass housing construction and urban growth, new construction has steadily declined to at present well below 1 % annually of the existing stock, to be replaced by a growing emphasis on maintenance, renewal and adaptation of the fast ageing existing stocks. In the same time, the also ageing population and the changing housing market came with changing demands and increasing spatial segregation, culminating in less wanted stocks and neighbourhoods. This paradigm shift had—and still has—far reaching consequences on a wide scale of participants, professions and skills. In spite of this, the knowledge in the broad field of housing renewal was up to recently only slowly disclosed and the number of comprehensive publications—apart from an abundancy of single-issue papers and mostly local language written case studies—is remarkably limited.

*Renewing Europe’s Housing* is up to date the first compendium covering the policy knowledge field of the existing housing stock and the way it is governed across Europe and that—as also emphasized by three renowned scholars on the backside blurb—accounts for its importance. The authors are known experts with a long list of papers on the issue, with as a consequence and due to the limited number of other references a rather high proportion of self-citations and earlier published content, albeit updated and reedited.

The book is structured around a series of nine country chapters that make up the body of the book, preceded by an introductory chapter and followed by two concluding chapters. The country chapters cover a series of comparative accounts of housing renewal in various European countries. They follow a similar structure: an introductory overview describing the character and the development of the national stock and underlying housing policies illustrated with statistical data, graphs etcetera, followed by some exemplary case studies and completed with a concluding section accounting the main physical, economic, social, cultural and community aspects and difficulties.

In the first concluding chapter, Tim Brown and Richard Turkington give a short but recognizable and useful overview of changing approaches to policy making in housing and urban renewal, partly taken from the country chapters, partly from other sources, from which they distil four influences on policy making that can be used as a checklist. The last concluding chapter summarizes the origins, the development and the present focus of housing renewal and looks at what has been achieved and what are the lessons learnt. Important conclusions are that despite variations between countries there is a wide degree of common ground about the nature of the problem and effective methods and techniques to deal with it, about the essential role of existing communities and thus area-based approaches important principles in housing renewal, and about the importance of sustainability gains and of evidence-based policies. Regarding the implementation of housing renewal, a typology of main approaches followed in all the countries involved is distinguished: area based; local involvement especially of residents; leadership and partnership; holistic coordination; prevention- and recourse-based strategies, followed by a threefold techniques to assess the effectiveness.

The main aims of the authors are (p. 3) “to provide contemporary accounts of housing policy and practice in nine European countries; to see how individual countries’ approaches develop over time; and to bring together information and ideas on how the renewal of older housing (…) can achieve more prominence in the policy agenda in Europe. In short (…) to raise the profile of housing renewal as a focus for policy”. This focus of the book is both its strength and its constraint.

The strength of the book is—as acknowledged above—the indisputable value as handbook on housing renewal that surely will be widely used and cited. The book does not so much present totally new findings or undisclosed views; able practitioners with an international network will not find many surprises. But the authors did assemble it all, and the fact that they succeeded in bringing together, unravel and structure the issue is quite an achievement. Take alone the term “housing renewal”. What the authors conclude as the “striking variety of ways the term is found to be used” can not only be explained by history, culture and semantics. It stands for the nature of this multifaceted, multi-actor, cross-linked, multi-impact, time and space depending wicked problem area. This nature is also the other side of the medal, as it can only be thoroughly approached in a multidisciplinary holistic way, and there lies the—maybe inevitable—major constraint of the study: the limited focus on policy and policy issues, in particular formal governmental, mainly state-based policies. That may be understandable regarding the policy directed main aim and to keep the focus feasible. But housing renewal is—howsoever defined—wider, as it is influenced by and acting upon housing and building markets, involving a multitude of interests from residents, users and tradesmen to planners, architects, real estate and property professionals, consultants, contractors et cetera and covering a wide area of knowledge fields, from real estate economy and property management, urban planning, architecture, urban and building pathology to health and social safety. On all those fields, a flow of contemporary studies regarding housing renewal is being published with also policy-relevant knowledge. Determining discussions about, for example, the choice of intervention, alternative approaches, planning strategies, renovation or demolition, investments and returns, sustainable solutions, hidden agendas, conflicting policies, all those aspects of the wicked problem character of housing renewal, cannot be understood nor tackled without some kind of connection to these knowledge sources.

Also in this respect, some of the country chapters, e.g. the Netherlands, are a bit single sided, focussing on post-war social housing, problem areas and larger-scale government-driven interventions while underexposing private stock areas and owner-occupied renewal. This may partly mirror the availability and accessibility of data, partly the fact that most authors have been previously cooperating on high-rise and large-scale problem areas.

Regarding the differences between the country chapters, the comparative analyses may possibly have gained from a more strictly defined format and sharper formulated research questions, but that is difficult to guess as the insight in the underlying research is limited.

In their conclusions, the authors point at some recent developments—the sub-prime crisis and the increasing urgency of the global climate change—that will have major consequences for the conditions, policies and practices of housing renewal. In the meantime, the recent stagnation of the economy showed in most western-European countries, a further shift towards existing stock investments. Not explicitly mentioned but not least important is the ongoing financialization of the economy that deeply affects the capital intensive property sector, with near bankruptcy of housing associations in the Netherlands and similar effects elsewhere in Europe.

*Renewing Europe’s Housing* is all together a broad comparative inventory of valuable data, insights and approaches and state-of-the-art analysis of housing renewal in Europe. It’s well-documented content about the importance of housing renewal contains strong arguments to attain its main aim: raising the attention for the growing importance of housing renewal as principal political assignment. But it’s probably longest lasting contribution to housing renewal is being an indispensable handbook for professionals and students.

